# Early Invasive Strategy for Unstable Angina: a New Meta-Analysis of Old Clinical Trials

**DOI:** 10.1038/srep27345

**Published:** 2016-06-07

**Authors:** Olivia Manfrini, Beatrice Ricci, Ada Dormi, Paolo Emilio Puddu, Edina Cenko, Raffaele Bugiardini

**Affiliations:** 1 Department of Experimental, Diagnostics and Specialty Medicine, University of Bologna, Via Giuseppe Massarenti 9, 40138 Bologna, Italy; 2 Department of Medical and Surgical Sciences, University of Bologna, Via S. Giacomo 12, 40126 Bologna, Italy; 3 Department of Cardiovascular, Respiratory, Nephrological, Anesthesiological and Geriatric Sciences, Sapienza University of Rome, Viale del Policlinico 155, 00161 Rome, Italy

## Abstract

Randomized controlled trials (RCTs) were conflicting to support whether unstable angina versus non-ST-elevation myocardial infarction (UA/NSTEMI) patients best undergo early invasive or a conservative revascularization strategy. RCTs with cardiac biomarkers, in MEDLINE, EMBASE, and Cochrane Central Register of Controlled Trials from 1975–2013 were reviewed considering all cause mortality, recurrent non-fatal myocardial infarction (MI) and their combination. Follow-up lasted from 6–24 months and the use of routine invasive strategy up to its end was associated with a significantly lower composite of all-cause mortality and recurrent non-fatal MI (Relative Risk [RR] 0.79; 95% confidence interval [CI], 0.70–0.90) in UA/NSTEMI. In NSTEMI, by the invasive strategy, there was no benefit (RR 1.19; 95% CI, 1.03–1.38). In the shorter time period, from randomization to discharge, a routine invasive strategy was associated with significantly higher odds of the combined end-point among UA/NSTEMI (RR 1.29; 95% CI, 1.05–1.58) and NSTEMI (RR 1.82; 95% CI, 1.34–2.48) patients. Therefore, in trials recruiting a large number of UA patients, by routine invasive strategy the largest benefit was seen, whereas in NSTEMI patients death and non-fatal MI were not lowered. Routine invasive treatment in UA patients is accordingly supported by the present study.

Two strategies have been used in managing patients with unstable angina/non-ST-elevation myocardial infarction (UA/NSTEMI). Patients may undergo an early invasive strategy of coronary angiography and revascularization by percutaneus coronary intervention (PCI) or a conservative, “ischemia guided” strategy in which hemodynamic procedures are performed only if there is evidence of recurrent ischemia or high risk features^1^.

Clinical trials offered conflicting evidence to support one strategy over the other. As a result, there was considerable interest in summating the available information from large-scale clinical trials by using meta-analyses and systematic reviews that can provide a more robust estimate of the effect of a specific therapy. Yet, even a number of meta-analyses have led to contradictory results regarding the efficacy of a routine use of an invasive strategy to reduce both nonfatal myocardial infarction (MI) and mortality^1^,^2^,^3^,^4^,^5^,^6^,^7^,^8^,^9^,^10^.

There are a number of potential limitations of these studies, mainly heterogeneity, i.e. the extent to which different trials may give similar or different results^1^,^2^,^3^,^4^,^5^,^6^,^7^,^8^,^9^,^10^. Although statistical tests are routinely available to evaluate heterogeneity, physicians are not interested in this and rather look at clinical heterogeneity, i.e., specific pathophysiologic causes that underlie heterogeneity across studies.

In the current analysis, we explored the hypothesis that trials including a substantial number of patients with UA may provide evidence of a reduced rate of death and/or recurrent MI when treatment was by an early invasive strategy as compared to trials recruiting participants with just NSTEMI.

To test this hypothesis we compared trials enrolling patients with both positive and negative biomarkers (UA/NSTEMI) with those only recruiting participants with positive cardiac biomarkers (NSTEMI).

## Results

**Figure 1:**
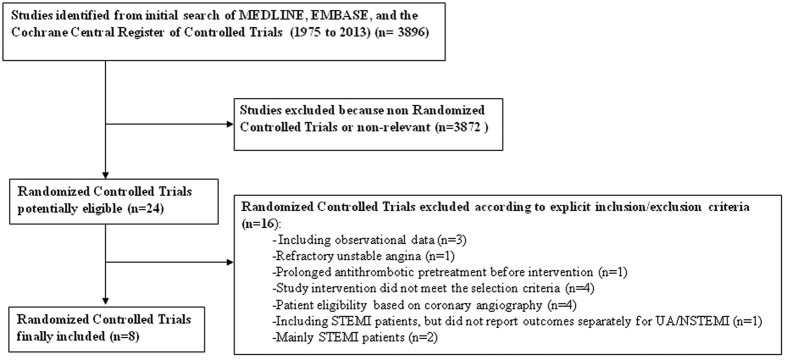
Flow diagram of search and selection.

The literature search yielded 3896 hits. From these, 24 studies were selected for closer attention. Of these 16 studies were excluded according to explicit inclusion/exclusion criteria^11^,^12^,^13^,^14^,^15^,^16^,^17^,^18^,^19^,^20^,^21^,^22^,^23^,^24^,^25^,^26^ (Fig. 1). The eight remaining studies satisfied the inclusion requirements of this analysis, namely: Effects of tissue plasminogen activator and a comparison of early invasive and conservative strategies in unstable angina and non-Q-wave myocardial infarction. Results of the Thrombolysis in Myocardial Ischemia (TIMI IIIB Trial)^27^, Results of the Medicine Versus Angiography in Thrombolytic Exclusion (MATE) Trial^28^, Veterans Affairs Non-Q-Wave Infarction Strategies in Hospital (VANQWISH) Trial^29^, FRagmin and Fast Revascularisation during In-Stability in Coronary artery disease (FRISC II) Trial^3^, Treat Angina with Aggrastat and Determine Cost of Therapy with an Invasive or Conservative Strategy (TACTICS-TIMI 18) trial^30^, Value of First Day Coronary Angiography/Angioplasty In Evolving Non ST-Segment Elevation Myocardial Infarction (VINO) Trial^31^, the Randomized Intervention Trial of unstable Angina 3 (RITA 3) randomized trial^32^ and Invasive versus Conservative Treatment in Unstable coronary Syndromes (ICTUS) Trial^2^ (Table 1).

### Baseline characteristics

Eight prospective randomized placebo-controlled clinical trials (RCTs) involving 10412 patients (range, 131 to 2457 patients per trial) were included in the analysis. The primary characteristics of the eight included trials are listed in Table 1. Patients were admitted to the hospital mainly because of UA/NSTEMI, and enrollment in the routine invasive intervention arm of the trial was completed within 98 hours of admission. Duration of the follow-up periods ranged from 6–24 months and few patients were lost to follow-up analysis. Some baseline patient clinical characteristics were different between the two randomized groups (NSTEMI versus UA/NSTEMI) of all the included studies (Table 1).

### Overall clinical outcomes at the end of follow-up

**Figure 2:**
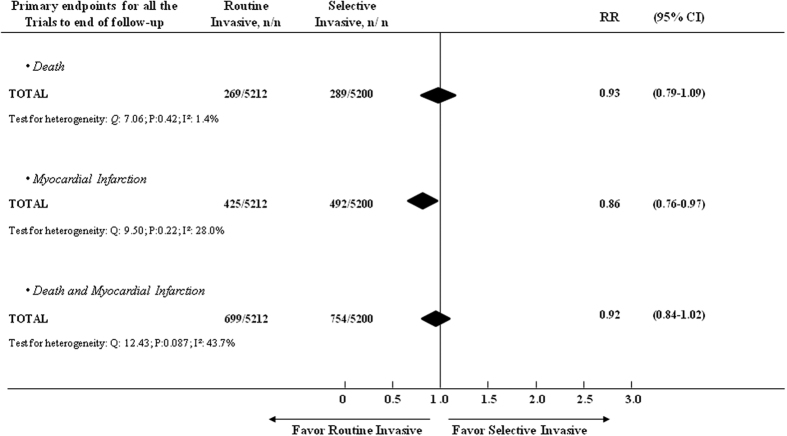
Primary end-points from randomization to end of follow-up for all the trials combined, comparing the efficacy of a routine vs. selective invasive strategy.

Compared with the conservative strategy, the OR for death with an invasive strategy was 0.93 (95% CI, 0.79–1.09), the OR for recurrent nonfatal MI was 0.86 (95% CI, 0.76–0.97), and the OR for death or MI was 0.92 (95% CI, 0.84–1.02) (Fig. 2).

### Outcomes for the two presentations

#### From randomization to the end of follow-up

**Figure 3:**
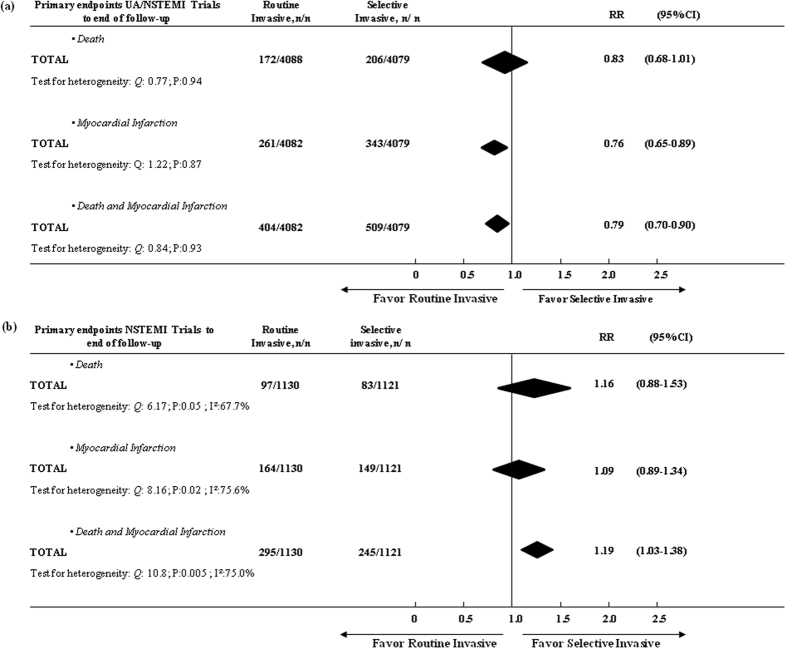
Comparing the efficacy of a routine vs. selective invasive strategy on primary end-points from randomization to end of follow-up in (**a**) UA/NSTEMI and (**b**) NSTEMI groups.

Based on the pooled results, the use of routine invasive strategy in studies that randomized UA/NSTEMI patients was associated with a dramatic reduction for the composite ischemic events with 20–30% lower odds based on the definitions of death and MI used in these trials (Fig. 3a). In contrast, there was no benefit of the use of such strategy (RR, 1.19; 95% CI, 1.03–1.38) in NSTEMI. The use of a selective invasive strategy in studies that only randomized NSTEMI patients was associated with moderate reductions in the risk for death and MI and with a significant effect on overall composite ischemic events (Fig. 3b). The observed effects were consistent among most evaluated trials except MATE trial among UA/NSTEMI studies and except VINO trials among NSTEMI studies.

#### From randomization to discharge

**Figure 4:**
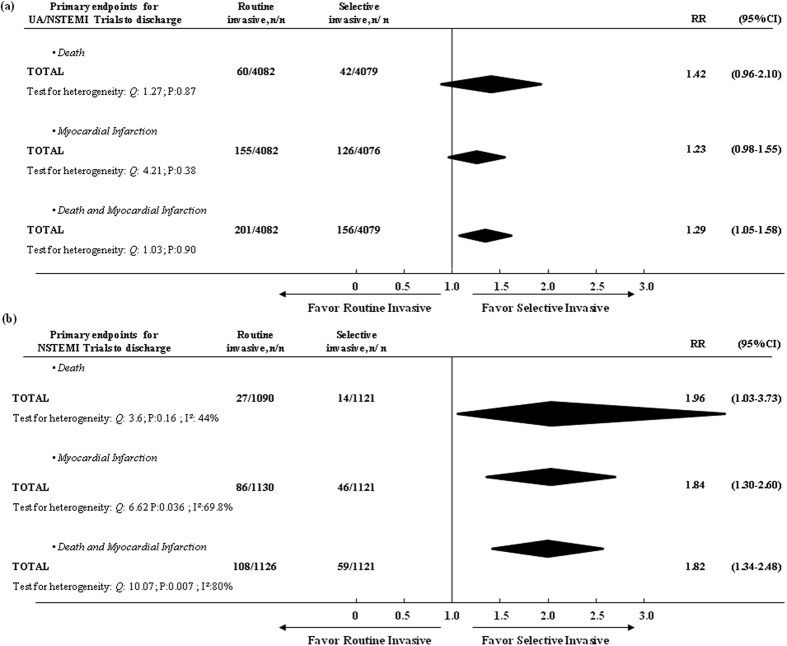
Comparing the efficacy of a routine vs. selective invasive strategy on primary end-points from randomization to discharge in (**a**) UA/NSTEMI and (**b**) NSTEMI groups.

A routine invasive strategy was associated with significantly higher odds of the combined endpoint in both UA/NSTEMI (RR, 1.29; 95% CI, 1.05–1.58) and NSTEMI (RR, 1.82; 95% CI, 1.34–2.48) (Fig. 4a,b). There was also a significant increase in the invasive arm of both the index death and MI (Fig. 4b).

#### Heterogeneity between trials

There was no evidence of heterogeneity among trials for the composite end points of death or MI (heterogeneity: *Q*:*12.43*, *P*:*0.087, I*^*2*^:*43.7%)*, death (heterogeneity: *Q*:7.06, P:0.42, I^2^:1.4%) or MI (heterogeneity: *Q*:9.50, P:0.22, I^2^:28.0%) when the period of time from randomization to the end of follow-up was considered (Fig. 2). In addition, there was no evidence of heterogeneity among UA/NSTEMI trials. Some heterogeneity was found among NSTEMI trials during time from randomization to discharge (Fig. 4b).

## Discussion

We found that a routine invasive strategy is of most benefit in trials recruiting a large number of UA patients, whereas it cannot be proven to reduce death or non-fatal MI among NSTEMI patients. Potential clinical benefits from PCI do not seem to favorably affect the overall prognosis of the index MI at a follow-up exceeding 1-year.

### The current meta-analysis

Eight RCTs published between 1975 and 2013 were included in this meta-analysis. Three of the five studies (VINO, VANQWISH and ICTUS) included only patients with elevated cardiac biomarkers; the other five studies (TIMI IIIB, MATE, FRISC-II, TACTIS-TIMI 18 and RITA-3) recruited participants with both positive and negative cardiac biomarkers. The percentage of biomarker-positive patients in the selected studies ranged between 17–58% (Table 1).

### Outcomes from randomization to the end of follow-up

Studies that only randomized biomarker-positive patients (NSTEMI) were analyzed separately and showed a 1.6-fold increase in the relative risk of death and MI in the invasive arm at a mean follow-up of 13.7 months. On the opposite, the composite end point was significantly decreased by an invasive strategy in those studies that did not require positive cardiac biomarker status as an inclusion criterion (UA/NSTEMI). The observed effects were consistent among most evaluated trials except MATE among UA/NSTEMI and VINO among NSTEMI trials, respectively.

### The exceptions

An exception to the potential hazards of an early invasive strategy in NSTEMI patients was the VINO trial. It should be noticed, however, that the VINO trial enrolled high-risk individuals with 53% of the patients having baseline Killip class II or III. Observational data have shown that Killip class II and III patients have significant mortality benefit from an invasive strategy while patients with Killip class I do not^33^,^34^. The high percentage of patients with heart failure in the VINO trial may explain, therefore, why a robust benefit for an early invasive strategy was found, despite the small cohort of NSTEMI.

An exception to the benefits of an early invasive strategy among trials recruiting a large number of UA patients was the MATE trial. Yet, in this trial, approximately 30% of the patients enrolled presented with acute ST-elevation myocardial infarction (STEMI) and only 23% of the study population had segment depression on the initial ECG. Thus, differently from the other UA/NSTEMI studies in our analysis, MATE randomized patients with borderline selection criteria, in whom the overall prognosis was partially affected by a late revascularization of the index MI. According to the Occluded Artery Trial no discernible benefit at four year follow-up was found among patients with occlusion of the infarct-related artery following a strategy of routine PCI late after acute MI^35^.

### Outcomes from randomization to discharge

Our data are concordant with prior doctrine that early or in-hospital coronary revascularization is fraught with hazard in patients with non-ST elevation acute coronary syndromes^5^,^36^. An updated meta-analysis of randomized trials showed that the routine invasive group had significantly more death or MI during the initial hospitalization compared with the selective conservative group^9^. In our study the primary outcome of death occurred in 1.7% of patients in the routine-intervention group and in 1% of those in the conservative strategy group. The difference was statistically significant among NSTEMI patients (RR, 1.96; 95% CI, 1.03–3.73) but not among UA/NSTEMI patients (RR, 1.42; 95% CI, 0.96–2.10). It is, therefore, reasonable to assume that overall there is an immediate hazard with any invasive procedure, which can be mitigated by the type of clinical presentation. The more severe is the disease, the greater is vulnerability. Patients with MI are known to be a more fragile group from the cardiovascular point of view than those with UA, and as so they have the greatest hazard^37^,^38^.

### Comparison with previous meta-analyses

Many old meta-analyses on this topic did not include the most recent trial, the ICTUS study, published in 2005^4^,^5^. More recent studies have shown that the invasive strategy had null effects on outcomes at 12 month follow-up^6^,^7^,^8^,^9^. The most recent meta-analysis^10^ of the only trials with long-term outcomes (FRISC-II, ICTUS, RITA-3) showed a sustained reduction in the rate of cardiovascular death or MI by using a routine interventional strategy only at 5 year follow-up.

The findings from our analysis differ substantially from those of previous studies, as an early invasive approach was not of benefit at a mean follow-up of 13.7 months in those trials that included only NSTEMI patients, but benefited those including UA patients.

### Unstable angina versus Non ST-Elevation Myocardial Infarction

Observations on the natural history of UA have shown an alarming incidence of death (6%) and the need for coronary revascularization by either PCI or coronary artery bypass graft in 27% of patients at 1-year follow-up^39^. Thereafter, the long-term outcome of UA is more favorable with a mortality rate of 2–3% in the following 7 years from hospital discharge. These findings stand in stark contrast to the low (2.5%), 1-year mortality reported from the ICTUS trial, where only NSTEMI patients were enrolled. Yet in this study, mortality rates at 5-year follow-up were much greater than those reported for UA patients, respectively at 11.1 and 9.9% after routine or selective invasive procedures^40^. These diverging results cannot be explained by differences in clinical practice. The different findings in studies enrolling UA/NSTEMI participants may, therefore, be due to differences in the natural history of UA/NSTEMI. The strength of a prognostic factor may be greater during one period of time than another, which may underlie different pathogenetic substrates and obscure the perception of the best therapeutic strategy. In this case, the potential clinical benefits from PCI do not seem to favorably affect the overall short-term prognosis of NSTEMI, which is mainly given by the amount of ventricular injury due to the index event.

### Benefits at short versus long-term follow up

Current literature indicates that an invasive strategy had favorable effect on mortality at 5 year follow-up^5^. It should be noted, however, that the risk of late adverse outcomes may be related to many factors^10^. Differences in patient selection and supportive care can lead to better outcomes late after PCI. Patients undergoing PCI are younger and more motivated. Thus, they are more likely to be adherent to medication prescriptions and are also more likely to participate in cardiac rehabilitation^41^. Earlier benefit of PCI, on the opposite, may support the plausibility of a true causal relationship. It takes a short time to demonstrate the potential of a new therapeutic strategy.

### Guidelines and take home message

When we performed a PubMed search in April 2013, we found no recent trials on the subject of invasive versus conservative treatment in patients with unstable coronary syndromes. The AHA/ACC treatment guidelines for UA/NSTEMI recommend (class I) invasive treatment in patients with UA/NSTEMI who have an elevated risk for clinical events as defined by risk scores and additional risk factors (e.g LVEF less than 40%, prior CABG and PCI within 6 months)^1^. On the other hand, the Task Force on the Management of Myocardial Revascularization of the ESC recently stated in their document that timing of angiography and revascularization should be based on patients risk profile^42^. The same guidelines recommend an early invasive strategy within 24 hours in patients with at least one primary high-risk criterion (relevant rise in troponin, dynamic ECG changes, and GRACE score > 140), but large randomized trials for these indications are lacking. The results of our study support recommendation for routine invasive treatment of UA patients, even when thay have stabilized after an acute coronary syndrome.

### Study limitations

We used the same trials included in the preceding meta-analyses that so far offer background to guidelines’ recommendations. There are limitations in the published meta-analyses^4^,^5^,^6^,^7^,^8^,^9^,^10^. Indeed there was heterogeneity in studies with different inclusion criteria, variable times of interventions and differential use of glycoprotein inhibitors. The same limitations apply to our study. As well, the current as the other studies investigated the impact of troponin assays on the outcomes of myocardial infarction before the advent of hs-troponins and the more recent definition of myocardial infarction^43^. The use of hs-troponins and implementation of a lower diagnostic threshold disproportionately increases the incidence of MI. Yet UA still exists and our findings support the use of a routine invasive strategy in this clinical condition. Given that the trials used in this meta-analysis are more consistent with a ’delayed invasive’ strategy, it is possible that the available data under-estimate the potential effectiveness of the invasive strategy^44^,^45^,^46^. Finally, the significance of peri-procedural myocardial infarction is a subject of considerable debate. A universal definition of myocardial infarction, including peri-procedural myocardial infarction, has been adopted only recently and defines peri-procedural myocardial infarction as elevated cTn values following procedure^43^,^47^. Unfortunately, the peri-procedural myocardial infarction was variably defined in the included studies and not for all studies (appendix table). However, end-points such as death are indisputable, and there was also a significant increase of death in the invasive arm.

## Conclusions

Today, the evidence base for the most appropriate treatment of UA/NSTEMI is still limited, and there are differences between the European and the American guidelines. The current study supports class I recommendation of routine invasive treatment for patients diagnosed as UA. These results emphasize the need for further randomized trials to provide a better evidence base for the invasive versus conservative treatment of patients after UA/NSTEMI.

## Methods

### Search strategy

We searched RCTs in English language, from 1975 to October 2013, through MEDLINE, EMBASE, and the Cochrane Central Register of Controlled Trials databases using the keywords: *acute coronary syndrome*, *unstable coronary syndrome*, *unstable angina*, *non-st-elevation myocardial infarction, early invasive therapy* and *selective invasive therapy.*

### Study characteristics

Eligible studies for inclusion were RCTs comparing a routine invasive versus a selective invasive strategy in patients presenting with UA/NSTEMI. Only studies reporting data on cardiac biomarkers were considered for inclusion. Trials were excluded if the majority of patients had STEMI or stable ischemic disease (Fig. 1).

### Outcome measures

The primary end-points were mortality, recurrent non-fatal MI and their combination identified in two periods of time: from randomization to discharge and from randomization to the end of follow-up. The follow-up was extracted from each original article and was calculated as the mean of all studies included. The mean follow-up was 16 months (range, 6–24).

### Data extraction an statistical analysis

Data were extracted independently by two authors of the present study onto data extraction sheets. Disagreement was resolved first by consensus and then by consultation with another two co-authors.

We analyzed trials recruiting participants with positive cardiac biomarkers versus those that did not specify cardiac biomarker status as an inclusion criterion.

To standardize the reporting of our results we calculated relative risk (RR) and 95% confidence intervals (CI) from multiple standard 2 × 2 tables built based on the number of events among the participants for each group in every trial. We used fixed effect model meta-analysis to assess the effect of selective invasive treatment versus routine invasive treatment on the outcomes of interest. The *Q* statistic and *I^2^* index were used to assess statistical heterogeneity across trials. The *Q* statistic was considered significant if the p < 0.10, and heterogeneity was considered high if the *I^2^* index >50%.

We also used a random effect model when the statistical test of heterogeneity showed significance. However, the results did not differ qualitatively from those of the primary analysis. Furthermore the results were double-checked performing analysis on the risk ratios’ log scale, using the log of the ratio and its corresponding standard error for each study, calculated according to Woolf’s method. Results were displayed as Forest Plots using fixed-effects summary estimates.

To examine the influence of individual studies on the summary effect estimate, we performed an influence analysis, in which the meta-analysis estimates are computed omitting one study at a time. Finally, to analyze associations between treatment effect and study characteristics we used meta-regression for the possible confounders. Analyses were performed using STATA software version 11 and all tests were 2-sided.

## Additional Information

**How to cite this article**: Manfrini, O. *et al.* Early Invasive Strategy for Unstable Angina: a New Meta-Analysis of Old Clinical Trials. *Sci. Rep.* **6**, 27345; doi: 10.1038/srep27345 (2016).

## Supplementary Material

Supplementary Appendix table1

## Figures and Tables

**Table 1:** Characteristics of Included Randomized Controlled Trials and Baseline Characteristic of Patients.

	Trial, Year (Reference)	Antithrombotic therapy and its duration	GPIIB/IIIA Inhibitor, %	Stenting invasive arm, %	Follow-up, mo	Participants, n	Mean Age, y	Men, n (%)	
UA/NSTEMI TRIALS	TIMI IIIB^29^	Aspirin, UFH IV heparin 72 to 96 h; ASA from 2° day for 1 year	0	NA	12	1473	59	972 (66.0)	
	MATE^30^	Aspirin, UFH Not specified	0	<50	21	201	59	129 (64.2)	
	FRISC-II^3^	Aspirin, Dalteparin Dalteparin at least 5 days; ASA not specified	10	61	24	2457	65	1708 (69.5)	
	TACTICS TIMI 18 32	Aspirin, UFH, Tirofiban ASA not specified; UFH 48 hours or until revascularization; Tirofiban at least 12 hours	94	83	6	2220	62	1443 (65.0)	
	RITA 3 34	Aspirin, Enoxaparin For 2–8 days	25	88	24	1810	63	1128 (62.3)	
	**Subtotal**	–	–	–	**17.4**	**8161**	**62 ± 9.0**	**5380** (**65.9**)	
NSTEMI TRIALS	VANQWISH^31^	Aspirin, UFH Not specified	0	0	23	920	61	896 (97.4)	
	VINO^33^	Aspirin, UFH UFHr at least 3 days; ASA the duration of the study	0	44	6	131	66	80 (61.1)	
	ICTUS^2^	Aspirin, UFH, Enoxaparin, Abciximab (Clopidogrel after 2002) ASA indefinitely; Enoxaparin at least 48 hours; Abciximab 12 hours	100	88	12	1200	62	880 (73.3)	
	**Subtotal**	**–**	**–**	**–**	**13.7**	**2251**	**62.3 ± 9.9**	**1856 (82.5)**	
UA/NSTEMI TRIALS	**Trial, Year (reference)**	**Prior MI, n (%)**	**Current smoker, n (%)**	**Diabetes mellitus, n (%)**	**Hypertension, n (%)**	**High cholesterol level, n (%)**	**ST-segment depression, n (%)**	**Rate of positive biomarkers** (**invasive/conservative**), **%**	**Rate of patients undergoing to Routine Invasive Strategy**
	TIMI IIIB^29^	604 (41.0)	545 (37.0)	NA	619 (42.0)	NA	486 (33.0)	34/30	50.2% Within 18–48 hours from randomization
	MATE^30^	43 (21.4)	41 (20.4)	36 (17.9)	NA	83 (41.3)	47 (23.4)	51/54	55.2% Within 24 hours of arrival to the hospital
	FRISC-II^3^	546 (22.2)	745 (30.3)	299 (12.2)	743 (30.3)	1397 (56.9)	1114 (45.4)	57/58	49.7% Within 7 days
	TACTIS TIMI 18 32	866 (39.0)	NA	613 (27.6)	NA	NA	688 (31.0)	56/52	50.2% Within 4 – 48 hours after randomization
	RITA 3^34^	501 (27.7)	586 (32.4)	244 (13.5)	632 (35.0)	579 (32.0)	660 (36.5)	19/17	49.4% Within 72 hours after randomization
NSTEMI TRIALS	**Subtotal**	**2560** (**31.4**)	**1917** (**32.3**)	**1192** (**17.8**)	**1994** (**34.7**)	**2059** (**46.1**)	**2995** (**36.7**)	–	–
	VANQWISH^31^	396 (43.0)	399 (43.4)	240 (26.1)	498 (54.1)	157 (17.1)	356 (38.7)	100	50.2% Within 24 to 72 hours after symptoms onset
	VINO^33^	34 (26.0)	NA	33 (25.2)	67 (54.1)	NA	60 (45.8)	100	48.9% Within 24 hours of last rest chest pain
	ICTUS^2^	278 (21.2)	492 (41.0)	166 (13.6)	466 (38.8)	417 (34.8)	574 (47.8)	100	50.3% Within 24 – 48 hours after randomization
	**Subtotal**	**708** (**31.4**)	**891** (**42.0**)	**439** (**19.5**)	**1031** (**45.8**)	**574** (**27.1**)	**990** (**44.0**)	–	–

TIMI IIIB, Thrombolysis in Myocardial Ischemia; MATE, Medicine versus Angioplasty for Thrombolytic Exclusions; FRISC-II, FRagmin and Fast Revascularization During InStability in Coronary Artery Disease II; TACTICS TIMI 18, Treat Angina With Aggrastat and Determine Cost of Therapy With Invasive or Conservative Strategy; RITA-3, Randomized Intervention Trial of Unstable Angina-3; VANQWISH, Veterans Affairs Non-Q-Wave Infarction Strategies in Hospital Trial; VINO, Value of First Day Coronary Angiography/Angioplasty In Evolving Non ST-Segment Elevation Myocardial Infarction Trial; ICTUS, Invasive versus Conservative Treatment in Unstable coronary Syndromes Trial; UFH, Unfrationated Heparin; GP, glycoprotein; MI, myocardial infarction; NA, data not available.
