# Supplementary material for: Early Invasive Strategy for Unstable Angina: a New Meta-Analysis of Old Clinical Trials
Source: Sci Rep. 2016 Jun 7;6:27345. doi: 10.1038/srep27345 (PMC4895177; doi:10.1038/srep27345)
Supplement: Supplementary Appendix table1 [file srep27345-s1.doc]

**SUPPLEMENTARY INFORMATION TO:**

**Early Invasive Strategy for Unstable Angina: a New Meta-Analysis of Old Clinical Trials.**

**Brief Title:** Invasive versus Conservative Strategy In UA/NSTEMI

Olivia Manfrini^1^; Beatrice Ricci^1^; Ada Dormi^2^; Paolo Emilio Puddu*^3^; Edina Cenko^1^; Raffaele Bugiardini^1^

**Author affiliations:**

1. Department of Experimental, Diagnostics and Specialty Medicine, University of Bologna, Via Giuseppe Massarenti 9, 42138 Bologna, Italy
2. Department of Medical and Surgical Sciences, University of Bologna, Via S. Giacomo 12, 40100 Bologna, Italy
3. Department of Cardiovascular, Respiratory, Nephrological, Anesthesiological and Geriatric Sciences, Sapienza University of Rome, Viale del Policlinico 155, 00161 Rome, Italy

**Word Counts:** 3020

***Corresponding author**: Paolo Emilio Puddu, MD, PhD, FESC, FACC.

Department of Cardiovascular, Respiratory, Nephrological, Anesthesiological and Geriatric Sciences, University of Rome “La Sapienza”. Viale del Policlinico, 155 Rome 00161 - Italy. E-mail: paoloemilio.puddu@uniroma1.it; telephone: +39-06 49972659 and fax: +39-06 4453891

**Appendix Table 1. (Definition of Procedural Myocardial Infarction in the Studies considered for this meta-analysis)**

**RITA-3.** Clinical symptoms, ECG changes and CK-MB or Toponin >2 x upper limit of normal greater than 24 hours post- randomization

**ICTUS.** CK-MB > upper limit of normal or a 50% decline from a peak value followed by subsequent rise to a value greater than the upper limit of normal.

**TACTICS TIMI 18.** CK-MB > 3 times upper limit of normal or >50% over previous.

**VINO.** Recurrent ischemic chest pain lasting >20 minutes, new ECG changes and CK-MB > 1.5 times the upper limit of normal after 72 hours post-randomization.

**FRISC-II.** Two or three of the following criteria: chest pain, ECG changes or elevated markers of myocardial damage: CK-MB mass > 1.5 times upper limit of normal or CK, CK-B, CK-MB activity > 3 times upper limit of normal in 1 sample of CK-MB activity > 2 times upper limit of normal in 2 samples.
